# Nitrate of Potash in the Cure of Intermittent Fever

**Published:** 1866-12

**Authors:** 


*Nitrate of Potash in the Cure of Intermittent Fever.*

In the St. Louis *Medical and Surgical Journal*, Amos Sawyer, M. D., of Hillsboro', Illinois, publishes the following formula:

℞. Potassæ nitrat., gr. x.
Spr. vini gallici, vel aquæ, f. ℥ ss. M.

Take immediately.

He says:—The above prescription I have used with great success in the cure of intermittent fever, even where quinine has failed. In my opinion, no preparation is equal to it; for it possesses anti-periodic properties completely, and may be administered when the stomach would not tolerate quinine. I deem it a specific in ague; for I have never failed to arrest the paroxysm, if uncomplicated. You will also find that the patients are less liable to relapse than in those cases cured by quinine. In the cold stage, if administered in a full dose, and the patient be placed in bed and covered with blankets, he will, in a few minutes, experience considerable heat, which will be followed by copious perspiration, and every unpleasant feeling will vanish. It is seldom the patient will experience a second attack. Where it is more agreeable to the patient, the powder may be placed on the tongue and permitted slowly to dissolve.

I shall not attempt to explain the action of this medicine on the system in the cure of ague, but will leave that to older heads than mine to determine; still, we do know that after it is taken into the stomach and becomes absorbed, it has the chemical effect of changing the dark-colored venous blood to arterial—or at least it changes its color. It also acts on the kidneys as a stimulant, producing diuresis as well as diaphoresis, and in this manner may rid the system of the poison that causes the ague; provided that poison is produced "by the retention of materials destined for excretion." This medicine more closely resembles nature's mode of curing this same disease than any other, as she cures by copious diaphoresis as well as diuresis, or, in other words, by elimination.

